# Cause of Death Among Patients With Thyroid Cancer: A Population-Based Study

**DOI:** 10.3389/fonc.2022.852347

**Published:** 2022-03-14

**Authors:** Qian Wang, Zhen Zeng, Junjie Nan, Yongqiang Zheng, Huanbing Liu

**Affiliations:** ^1^ Department of General Practice, The First Affiliated Hospital of Nanchang University, Nanchang, China; ^2^ Department of Geriatric Medicine, The First Affiliated Hospital of Nanchang University, Nanchang, China; ^3^ Cancer Center, Union Hospital, Tongji Medical College, Huazhong University of Science and Technology, Wuhan, China; ^4^ Zhejiang Provincial Key Laboratory of Laparoscopic Technology, Sir Run Run Shaw Hospital, School of Medicine, Zhejiang University, Hangzhou, China; ^5^ State Key Laboratory of Oncology in South China, Sun Yat-sen University Cancer Center, Guangzhou, China

**Keywords:** thyroid cancer (TC), cause of death, epidemiology, population-based study, SEER (Surveillance Epidemiology and End Results) database

## Abstract

**Background:**

Over the last decades, the number of patients diagnosed with thyroid carcinoma has been increasing, highlighting the importance of comprehensively evaluating causes of death among these patients. This study aimed to comprehensively characterize the risk of death and causes of death in patients with thyroid carcinoma.

**Methods:**

A total of 183,641 patients diagnosed with an index thyroid tumor were identified from the Surveillance, Epidemiology, and End Result database (1975–2016). Standardized mortality rates (SMRs) for non-cancer deaths were calculated to evaluate mortality risk and to compare mortality risks with the cancer-free US population. Cumulative mortality rates were calculated to explore the factors associated with higher risk of deaths.

**Results:**

There were 22,386 deaths recorded during follow-up, of which only 31.0% were due to thyroid cancer and 46.4% due to non-cancer causes. Non-cancer mortality risk among patients with thyroid cancer was nearly 1.6-fold (SMR=1.59) that of the general population. Cardiovascular diseases were the leading cause of non-cancer deaths, accounting for 21.3% of all deaths in thyroid cancer patients. Non-cancer causes were the dominant cause of death in thyroid cancer survivors as of the third year post-diagnosis. We found that males with thyroid cancer had a higher risk of all-cause mortality compared with females. The risk of suicide was highest in the first post-diagnostic year (<1 year: SMR=1.51). The long-term risk of Alzheimer’s disease was notably increased in thyroid cancer patients (>5 years: SMR=8.27).

**Conclusion:**

Non-cancer comorbidities have become the major risks of death in patients with thyroid tumor in the US, as opposed to death from the tumor itself. Clinicians and researchers should be aware of these risk trends in order to conduct timely intervention strategies.

## Introduction

Global morbidity due to thyroid tumors has risen notably in the last three decades (1). There were approximately 586,202 incident thyroid cancer cases and 43,646 thyroid cancer deaths worldwide in 2020 (2). This increasing incidence has been referred to as an epidemic of increasing surveillance and overdiagnosis; however, research indicates that this might also reflect a real increase in new cases of thyroid tumor (3–5). Due to the tremendous progress in cancer screening techniques and the increasing popularization of routine physical examinations in recent decades, a larger number of subclinical lesions have been detected. This has contributed to the high prevalence of low-risk, non-lethal tumors.

Improvements in the detection of thyroid tumors and therapeutic strategies have likewise resulted in favorable prognoses and low mortality rates for this disease (5). Hence, disease morbidity is incomparably higher than mortality for thyroid cancer and the population of cancer survivors continues to grow. However, although this disease presents with a relatively low mortality rate, the social burden caused by thyroid cancer has increased sharply. In addition to the direct and indirect medical, financial, and social costs of thyroid cancer, the long-term impacts of tumors may be multifaceted and complex. Thus, medical and epidemiologic research is necessary to elucidate the comprehensive medical and social costs of this disease.

One important change brought about by the rising population of cancer survivors is the increasing prevalence of deaths from causes other than the index carcinoma, including subsequent second or multiple primary cancers (6) and non-cancer comorbidities (7, 8). Some non-cancer comorbidities, including cardiovascular diseases (9, 10), infectious diseases (11), accidents (12), peptic ulcers (13), as well as suicides (14), have been reported as major threats to cancer patients’ health, survivorship, and quality of life. In addition, the risk of non-cancer comorbidities is increased as a consequence of the malignancy itself as well as the treatments administered. For example, an increased risk of non-cancer death immediately after cancer diagnosis has been reported (15). Although a few studies have evaluated the causes of death amongst patients with thyroid carcinoma (including Korea and Taiwan), the range of causes of death evaluated in these studies was much less comprehensive than in the current study and the sample sizes were small (16, 17). Moreover, the scope of these studies was limited (i.e., only a few common causes of death were evaluated). A comprehensive evaluation of the causes of death in patients with thyroid carcinoma is urgently needed. This will help researchers and clinicians identify patients at high risk of death and the particular causes of death for these patients.

The goal of this study was to comprehensively describe the distribution of cause of death in thyroid cancer patients with the purpose of providing constructive evidence for the health management of patients with thyroid tumors as well as optimization of their survival and quality of life.

## Materials And Methods

### Study Population and Data Source

This retrospective cohort study was conducted using data from the Surveillance, Epidemiology, and End Results (SEER) program, covering approximately 48.0% of the US population. Spanning over four decades, the SEER program routinely collects individual data on demographics, tumor morphology, stage at diagnosis, anatomic site, therapeutic modalities, and follow-up data for a range of cancer patients (18). In addition, due to its population-based program design, data from the SEER program can be used for comparisons with the general population, and thus are viable for estimating cancer incidence, mortality, and survival (19).

All thyroid cancer patients (site code C73.9) detected between 1975 and 2016 were retrieved from the SEER 18 database (i.e., the 2018 submission) (20). Only patients with an index thyroid malignancy were included in this study. Patients whose diagnoses were extracted from their death certificates and whose follow-up information was incomplete were excluded (see **Figure S1** for details). For comparison, we extracted age-, sex-, race-, and year-specific death data for the general US population between 1975 and 2016 (21, 22).

Since the SEER is a publicly available database, access to the data required a signed research data agreement form. Institutional review board approval and the need for informed consent were waived for data obtained from the SEER database, as the study did not involve new experiments on human subjects and all data were anonymized.

### Study Variables

For all patients, follow-up began at the time that the thyroid malignancy was detected and ended at the time of death from various causes, the finish of the research period (December 31, 2016), or due to early withdrawal. We extracted patients’ demographic and clinical information, including age, sex, race, marital status, age at diagnosis, survival time, stage at diagnosis, and therapy modality (i.e., surgery, radiotherapy, or chemotherapy), from the SEER database. Causes of death were classified into three chief categories: the index (thyroid) cancer, a non-index cancer (i.e., secondary or subsequent primary cancers), and non-cancer causes of death. According to the SEER cause-specific death classification variables evaluated herein, non-cancer deaths were classified into 26 categories (23, 24). Non-cancer deaths were additionally classified into seven major categories: cardiovascular disease, infectious disease, respiratory disease, kidney disease, gastrointestinal disease, external injury, and other causes. Deaths from tumors with a histology of “*in situ*, benign, or unknown behavior neoplasm” were not considered non-cancer deaths in our study. For patients who died within 1 month post-diagnosis, the SEER project recorded their survival months as 0 months. Given that the survival time of this category of patients varied from 0 to 29 days, we converted their survival months to the median of this duration (namely, 0.5 months).

### Statistical Analysis

We calculated mortality risks due to the index cancer, non-index cancers, and non-cancer causes of death in patients with thyroid carcinoma. For non-cancer causes of death, standardized mortality ratios (SMRs) were assumed to be the ratio of the death toll for patients with thyroid carcinoma to that of the general population. The calculation method for SMRs and associated 95% confidence intervals (CIs) with respect to non-cancer causes of death was as previously described (11, 25–27). SMRs were calculated as the ratio of the observed number of deaths in cancer patients to the expected number of deaths in the cancer-free population, which had a similar demographic structure in terms of sex, age, race, and the calendar year of study evaluations. For age and years of cancer diagnosis, the values obtained were used herein when the patients are detected with malignancy, and 5-year categories were created in the course of standardization. Associated 95% CIs were calculated using a Poisson distribution (25, 28). SMRs were not available for cancer-associated deaths, since SMRs are calculated based on an assumption that regards the general population as a cancer-free cohort (8). Based on this assumption, the cancer-free general population will not die from cancer; thus, the cancer-associated mortality in this population could not be estimated. The cumulative mortality rate (CMR) was calculated to determine those patients with thyroid carcinoma who were at higher risk of death (11). The JoinPoint model was used to calculated the annual percentage change (APC) of deaths from different causes, and test the statistical significance of trends in cause of death among thyroid cancer patients. An average APC was further calculated to summarize the trend over the entire time period (29).

R statistical software (30) (version 3.52; The R Project for Statistical Computing, Vienna, Austria) and SEER*Stat software version 8.3.6 (21) (National Cancer Institute, Bethesda, MD, USA) were used for all data analyses. All hypothesis tests were two-sided, with a P value of <0.05 set as the level of statistical significance.

## Results

Our study retrospectively reviewed and analyzed 183,641 US patients, diagnosed with an index thyroid carcinoma, with a median follow-up time of 6.9 years (range: 0–41.9 years). The majority of thyroid cancer patients were diagnosed at ages 40–59 years (44.1%). Female patients remarkably outnumbered male patients (76.6% *vs.* 23.4%). A majority of thyroid malignancies were detected before progressing to an advanced stage (localized, 55.8%; regional, 31.5%), while only a few thyroid cancer patients were diagnosed with advanced-stage disease (distant, 4.1%). Both surgery and radiotherapy are common treatments for thyroid cancer. In this population, the surgery rate was 95.7% and radiotherapy rate was 45.8%, while chemotherapy was less frequently applied (1.0%) (**Table 1**).

**Table 1:** Characteristics of patients diagnosed with first primary thyroid cancer from 1975 to 2016 in the SEER program.

Characteristics	No. of patients (%)	Total follow-up time (person-years)	No. of deaths (%)	Non-cancer deaths		
				No. of observed deaths (%)	No. of expected deaths (%)	SMR (95% CI)
All	183,641 (100.0%)	1,636,233	22,386 (100.0%)	10,387 (100.0%)	6,530.5	1.59 (1.56-1.62)
Age						
0-19	4,212 (2.3%)	49,098	89 (0.4%)	43 (0.4%)	19.5	2.21 (1.64-2.98)
20-39	54,971 (29.9%)	602,516	1,521 (6.8%)	814 (7.8%)	466.8	1.74 (1.63-1.87)
40-59	80,946 (44.1%)	710,440	6,751 (30.2%)	3,026 (29.1%)	1,728.0	1.75 (1.69-1.81)
60-79	38,622 (21.0%)	256,000	10,739 (48.0%)	5,030 (48.4%)	3,042.1	1.65 (1.61-1.70)
80+	4,890 (2.7%)	18,178	3,286 (14.7%)	1,474 (14.2%)	1,274.1	1.16 (1.10-1.22)
Sex						
Female	140,744 (76.6%)	1,270,557	14,208 (63.5%)	6,787 (65.3%)	4,110.8	1.65 (1.61-1.69)
Male	42,897 (23.4%)	365,676	8,178 (36.5%)	3,600 (34.7%)	2,419.6	1.49 (1.44-1.54)
Race						
White	148,868 (81.1%)	1,350,401	18,272 (81.6%)	8,507 (81.9%)	5,477.6	1.55 (1.52-1.59)
Black	12,058 (6.6%)	95,546	1,819 (8.1%)	941 (9.1%)	594.9	1.58 (1.48-1.69)
Other	22,715 (12.4%)	190,286	2,295 (10.3%)	939 (9.0%)	458.0	2.05 (1.92-2.19)
Year of diagnosis						
1975-1989	13,292 (7.2%)	333,786	5,672 (25.3%)	3,004 (28.9%)	1,039.4	2.89 (2.79-3.00)
1990-1999	18,643 (10.2%)	327,165	4,745 (21.2%)	2,205 (21.2%)	1,161.0	1.90 (1.82-1.98)
2000-2009	72,984 (39.7%)	735,679	8,951 (40.0%)	4,115 (39.6%)	3,184.9	1.29 (1.25-1.33)
2010-2016	78,722 (42.9%)	239,602	3,018 (13.5%)	1,063 (10.2%)	1,145.1	0.93 (0.87-0.99)
Marital status						
Married	112,649 (61.3%)	1,050,292	12,467 (55.7%)	5,606 (54.0%)	3,913.2	1.43 (1.40-1.47)
Unmarried	61,646 (33.6%)	521,789	9,034 (40.4%)	4,333 (41.7%)	2,344.9	1.85 (1.79-1.90)
Unknown	9,346 (5.1%)	64,152	885 (4.0%)	448 (4.3%)	272.3	1.65 (1.50-1.80)
Stage						
*In situ*	85 (0.05%)	1,127	14 (0.06%)	9 (0.1%)	3.9	2.28 (1.19-4.38)
Localized	102,400 (55.8%)	998,109	9,288 (41.5%)	6,006 (57.8%)	3,895.5	1.54 (1.50-1.58)
Regional	57,935 (31.5%)	547,367	7,711 (34.4%)	3,266 (31.4%)	2,121.5	1.54 (1.49-1.59)
Distant	7,442 (4.1%)	44,710	4,104 (18.3%)	629 (6.1%)	272.3	2.31 (2.14-2.50)
Unstaged	15,779 (8.6%)	44,919	1,269 (5.7%)	477 (4.6%)	237.2	2.01 (1.84-2.20)
Surgery						
Yes	175,800 (95.7%)	1,599,400	18,607 (83.1%)	9,391 (90.4%)	6,196.7	1.52 (1.49-1.55)
No	7,043 (3.8%)	31,686	3,562 (15.9%)	934 (9.0%)	301.4	3.10 (2.91-3.30)
Unknown	798 (0.4%)	5,147	217 (1.0%)	62 (0.6%)	32.4	1.92 (1.49-2.46)
Radiotherapy						
Yes	84,122 (45.8%)	728,512	9,503 (42.5%)	3,420 (32.9%)	2,660.1	1.29 (1.24-1.33)
No/Unknown	99,519 (54.2%)	907,721	12,883 (57.5%)	6,967 (67.1%)	3,870.4	1.80 (1.76-1.84)
Chemotherapy						
Yes	1,891 (1.0%)	6,948	1,361 (6.1%)	101 (1.0%)	44.7	2.26 (1.86-2.75)
No/Unknown	181,750 (99.0%)	1,629,285	21,025 (93.9%)	10,286 (99.0%)	6,485.8	1.59 (1.56-1.62)

### Causes of Death Among Patients With Thyroid Carcinoma

There were 22,386 individuals in total who died during follow-up, contributing to an all-cause mortality rate of 13.68.1 per 100,000 person-years. Among all deaths, 46.4% died from non-cancer causes (n=10,387, mortality rate: 634.8 per 100,000 person-years), 31.0% died from thyroid cancer (n=6,936, mortality rate: 423.9 per 100,000 person-years), and 22.6% died from other cancers (n=5,063, mortality rate: 309.4 per 100,000 person-years) (**Tables 1**, **2**). Cardiovascular disease caused the majority of non-cancer deaths (21.3%), followed by infectious diseases (3.3%) and external injuries (3.3%).

**Table 2:** Cause of death among patients with thyroid cancer in the SEER 18 program.

Cause of death	Cancer population		General population		SMR (95% CI)
	No. of observed deaths (%)	Mortality rates (per 100,000 person-years)	No. of expected deaths (%)	Mortality rates (per 100,000 person-years)	
**All causes**	22,386 (100.0%)	1368.1	NA	NA	NA
**Index cancer**	6,936 (31.0%)	423.9	NA	NA	NA
**Non-index cancer**	5,063 (22.6%)	309.4	NA	NA	NA
**Noncancer cause of death**	10,387 (46.4%)	634.8	6,530.5	399.1	1.59 (1.56-1.62)
**Infectious diseases**	735 (3.3%)	44.9	441.0	26.9	1.67 (1.55-1.79)
Pneumonia and influenza	366 (1.6%)	22.4	185.0	11.3	1.98 (1.79-2.19)
Syphilis	0 (0.0%)	0.0	0.1	0.005	0.00 (NA)
Tuberculosis	5 (0.02%)	0.3	5.2	0.3	0.97 (0.40-2.33)
Septicemia	209 (0.9%)	12.8	111.4	6.8	1.88 (1.64-2.15)
Other infectious diseases	155 (0.7%)	9.5	138.6	8.5	1.12 (0.96-1.31)
**Cardiovascular diseases**	4,767 (21.3%)	291.3	2,994.9	183.0	1.59 (1.55-1.64)
Diseases of heart	3,554 (15.9%)	217.2	2,313.8	141.4	1.54 (1.49-1.59)
Hypertension without heart disease	152 (0.7%)	9.3	63.1	3.9	2.41 (2.06-2.83)
Aortic aneurysm and dissection	76 (0.3%)	4.6	50.0	3.1	1.52 (1.21-1.90)
Atherosclerosis	63 (0.3%)	3.9	32.0	2.0	1.97 (1.54-2.52)
Cerebrovascular diseases	880 (3.9%)	53.8	501.3	30.6	1.76 (1.64-1.88)
Other diseases of arteries, arterioles, capillaries	42 (0.2%)	2.6	33.9	2.1	1.24 (0.92-1.68)
**Respiratory diseases**	608 (2.7%)	37.2	459.1	28.1	1.32 (1.22-1.43)
Chronic obstructive pulmonary disease and allied Cond	608 (2.7%)	37.2	459.1	28.1	1.32 (1.22-1.43)
**Gastrointestinal diseases**	176 (0.8%)	10.8	200.3	12.2	0.88 (0.76-1.02)
Stomach and duodenal ulcers	18 (0.08%)	1.1	16.7	1.0	1.08 (0.68-1.71)
Chronic liver disease and cirrhosis	158 (0.7%)	9.7	183.4	11.2	0.86 (0.74-1.01)
**Renal diseases**	301 (1.3%)	18.4	127.1	7.8	2.37 (2.11-2.65)
Nephritis, nephrotic syndrome and nephrosis	301 (1.3%)	18.4	127.1	7.8	2.37 (2.11-2.65)
**External injuries**	736 (3.3%)	45.0	739.6	45.2	1.00 (0.93-1.07)
Accidents and adverse effects	540 (2.4%)	33.0	491.8	30.1	1.10 (1.01-1.19)
Suicide and self-inflicted injury	164 (0.7%)	10.0	175.4	10.7	0.93 (0.80-1.09)
Homicide and legal intervention	32 (0.14%)	2.0	72.3	4.4	0.44 (0.31-0.63)
**Other cause of death**	3,064 (13.7%)	187.3	1,568.3	95.8	1.95 (1.89-2.02)
Alzheimers (ICD-9 and 10 only)	341 (1.5%)	20.8	115.2	7.0	2.96 (2.66-3.29)
Diabetes mellitus	482 (2.2%)	29.5	290.2	17.7	1.66 (1.52-1.82)
Congenital anomalies	19 (0.08%)	1.2	23.6	1.4	0.81 (0.51-1.26)
Certain conditions originating in perinatal period	2 (0.01%)	0.1	0.1	0.007	16.4 (4.11-65.8)
Complications of pregnancy, childbirth, puerperium	5 (0.02%)	0.3	4.7	0.3	1.07 (0.44-2.56)
Symptoms, signs and ill-defined conditions	166 (0.7%)	10.1	102.9	6.3	1.61 (1.39-1.88)
Other cause of death	2,049 (9.2%)	125.2	1,030.9	63.0	1.99 (1.90-2.08)

NA, not applicable.

Compared with the cancer-free population, the risk of mortality due to any type of non-cancer comorbidity was significantly greater in patients with a cancer history (SMR: 1.59; 95% CI: 1.56–1.62). Moreover, the risk of mortality due to particular non-cancer causes, including Alzheimer’s disease (SMR: 2.96; 95% CI: 2.66–3.29), hypertension (SMR: 2.41; 95% CI: 2.06–2.83), and renal disease (SMR: 2.37; 95% CI: 2.11–2.65) was significantly greater in patients with a cancer history (**Table 2**).

### Trends in Cause of Death Among Patients With Thyroid Carcinoma

**Figure 1:**
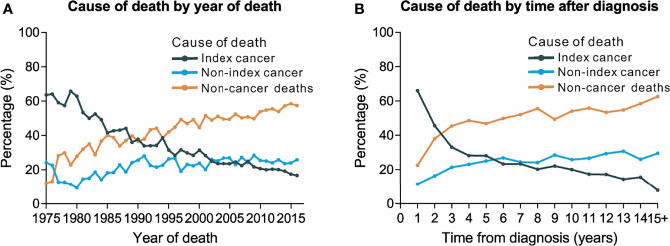
Trends of cause of death by year of death and time after diagnosis. **(A)** Trends of cause of death by year of death among patients with thyroid cancer. **(B)** Trends of cause of death by time after diagnosis.

We observed a rising trend in the proportion of non-cancer deaths in patients with thyroid carcinomas diagnosed between 1975 and 2016, whereas the proportion of deaths caused by thyroid cancer showed a rapid downward trend. More specifically, thyroid malignancy became the most common cause of death amongst patients with carcinoma thyroid malignancy between 1975 and 1990, while non-cancer causes were the major causes of death for persons with a history of thyroid malignancy between 1990 and 2016. The number of deaths from non-index cancers increased over this time (**Figure 1A**).

We further used JoinPoint model to test the trends in cause of death among thyroid cancer patients (**Figure S2**). The index-cancer deaths remained stable from 1975 to 1980 (APC=-0.64, p=0.6), decreased 6.24% annually from 1981 to 1985 (APC=-6.24, p<0.001), and then decreased 2.41% annually from 1985 to 2016 (APC=-2.41, p<0.001). This resulted in an average APC of -3.05 (p<0.001). Non-cancer deaths increased without statistical significance from 1975 to 1977 (APC=66.0, p=0.09), increased 2.48% annually from 1977 to 1997 (APC=2.48, p<0.001), and increased 1.09% annually from 1997 to 2016 (APC=1.09, p<0.001). This resulted in an average APC of 4.26 (p<0.001) (**Figure S2**).

A total of 21.8% (n=4,890) of deaths occurred within the first post-diagnostic year, 24.0% (n=5,369) occurred within 1–5 years after cancer diagnosis, and 54.2% (n=12,128) occurred more than 5 years post-diagnosis (**Table 3**). There was an increasing trend in the proportion of non-cancer deaths in patients with thyroid carcinoma over time (i.e., years after diagnosis), while the number of individuals who died from thyroid malignancy showed a rapid downward trend. Thyroid tumor has become the dominant cause of death in persons with thyroid malignancy within the first few years after diagnosis. However, as of the third post-diagnostic year, non-cancer causes became the dominant causes of death in survivors with a history of thyroid malignancy (**Figure 1B**). Non-cancer comorbidities were responsible for most deaths among long-term cancer survivors (**Figure 1B**). Within the first post-diagnostic year, 66.1% of deaths were attributed to thyroid cancer, whereas 22.5% were attributed to non-cancer causes. For persons who had a cancer history of more than 5 years, only 15.2% died from thyroid malignancy, while 57% of deaths were attributed to non-cancer causes (**Table 3**).

**Table 3:** Cause of death among patients with thyroid cancer by time after diagnosis.

Cause of deaths	Time after cancer diagnosis					
	With 12 months		12 to 60 months		More than 60 months	
	No. of deaths (%)	SMR (95% CI)	No. of deaths (%)	SMR (95% CI)	No. of deaths (%)	SMR (95% CI)
**All causes**	4,890 (100.0%)	NA	5,369 (100.0%)	NA	12,128 (100.0%)	NA
**Index cancer**	3,230 (66.1%)	NA	1,863 (34.7%)	NA	1,843 (15.2%)	NA
**Non-index cancer**	559 (11.4%)	NA	1,129 (21.0%)	NA	3,375 (27.8%)	NA
**Noncancer cause of death**	1,101 (22.5%)	1.31 (1.24-1.39)	2,376 (44.3%)	0.90 (0.87-0.94)	6,910 (57.0%)	2.26 (2.21-2.32)
**Infectious diseases**	90 (1.8%)	1.60 (1.30-1.96)	176 (3.3%)	0.99 (0.85-1.15)	469 (3.9%)	2.27 (2.07-2.49)
Pneumonia and influenza	42 (0.9%)	1.65 (1.22-2.24)	78 (1.5%)	1.01 (0.81-1.27)	246 (2.0%)	2.97 (2.62-3.37)
Syphilis	0 (0.0%)	0.00 (0.00-NA)	0 (0.0%)	0.00 (0.00-NA)	0 (0.0%)	0.00 (0.00-NA)
Tuberculosis	0 (0.0%)	0.00 (0.00-NA)	2 (0.04%)	1.21 (0.30-4.84)	3 (0.02%)	0.99 (0.32-3.08)
Septicemia	23 (0.5%)	1.46 (0.97-2.19)	57 (1.1%)	1.16 (0.89-1.50)	129 (1.1%)	2.77 (2.33-3.30)
Other infectious diseases	25 (0.5%)	1.70 (1.15-2.52)	39 (0.7%)	0.78 (0.57-1.06)	91 (0.8%)	1.23 (1.00-1.52)
**Cardiovascular diseases**	526 (10.8%)	1.38 (1.26-1.50)	1,088 (20.3%)	0.91 (0.86-0.97)	3,153 (26.0%)	2.23 (2.15-2.30)
Diseases of heart	390 (8.0%)	1.34 (1.21-1.48)	835 (15.6%)	0.91 (0.85-0.97)	2,329 (19.2%)	2.11 (2.02-2.20)
Hypertension without heart disease	17 (0.3%)	1.74 (1.08-2.79)	33 (0.6%)	1.14 (0.81-1.60)	102 (0.8%)	4.21 (3.47-5.11)
Aortic aneurysm and dissection	22 (0.4%)	3.66 (2.41-5.56)	16 (0.3%)	0.82 (0.50-1.33)	38 (0.3%)	1.56 (1.13-2.14)
Atherosclerosis	10 (0.2%)	2.24 (1.21-4.17)	14 (0.3%)	1.06 (0.63-1.79)	39 (0.3%)	2.72 (1.98-3.72)
Cerebrovascular diseases	81 (1.7%)	1.24 (0.99-1.54)	182 (3.4%)	0.90 (0.77-1.04)	617 (5.1%)	2.65 (2.45-2.87)
Other diseases of arteries, arterioles, capillaries	6 (0.1%)	1.40 (0.63-3.12)	8 (0.1%)	0.59 (0.30-1.19)	28 (0.2%)	1.74 (1.20-2.52)
**Respiratory diseases**	60 (1.2%)	0.95 (0.74-1.22)	137 (2.6%)	0.69 (0.58-0.82)	411 (3.4%)	2.08 (1.89-2.29)
Chronic obstructive pulmonary disease and allied Cond	60 (1.2%)	0.95 (0.74-1.22)	137 (2.6%)	0.69 (0.58-0.82)	411 (3.4%)	2.08 (1.89-2.29)
**Gastrointestinal diseases**	18 (0.4%)	0.81 (0.51-1.29)	44 (0.8%)	0.60 (0.44-0.80)	114 (0.9%)	1.09 (0.91-1.31)
Stomach and duodenal ulcers	1 (0.02%)	0.52 (0.07-3.67)	4 (0.07%)	0.64 (0.24-1.71)	13 (0.1%)	1.53 (0.89-2.63)
Chronic liver disease and cirrhosis	17 (0.3%)	0.84 (0.52-1.35)	40 (0.7%)	0.59 (0.43-0.81)	101 (0.8%)	1.06 (0.87-1.28)
**Renal diseases**	36 (0.7%)	1.96 (1.41-2.72)	68 (1.3%)	1.21 (0.95-1.53)	197 (1.6%)	3.75 (3.26-4.31)
Nephritis, nephrotic syndrome and nephrosis	36 (0.7%)	1.96 (1.41-2.72)	68 (1.3%)	1.21 (0.95-1.53)	197 (1.6%)	3.75 (3.26-4.31)
**External injuries**	84 (1.7%)	1.08 (0.87-1.33)	215 (4.0%)	0.82 (0.72-0.94)	437 (3.6%)	1.09 (1.00-1.20)
Accidents and adverse effects	53 (1.1%)	0.97 (0.74-1.27)	152 (2.8%)	0.84 (0.72-0.99)	335 (2.8%)	1.30 (1.17-1.45)
Suicide and self-inflicted injury	27 (0.6%)	1.51 (1.04-2.20)	45 (0.8%)	0.74 (0.55-0.99)	92 (0.8%)	0.95 (0.78-1.17)
Homicide and legal intervention	4 (0.1%)	0.70 (0.26-1.85)	18 (0.3%)	0.86 (0.54-1.37)	10 (0.08%)	0.22 (0.12-0.41)
**Other cause of death**	287 (5.9%)	1.31 (1.16-1.47)	648 (12.1%)	0.97 (0.89-1.04)	2,129 (17.6%)	3.14 (3.01-3.28)
Alzheimers (ICD-9 and 10 only)	8 (0.2%)	0.37 (0.19-0.75)	43 (0.8%)	0.73 (0.54-0.99)	290 (2.4%)	8.27 (7.37-9.28)
Diabetes mellitus	39 (0.8%)	1.05 (0.77-1.44)	113 (2.1%)	0.95 (0.79-1.14)	330 (2.7%)	2.46 (2.21-2.74)
Congenital anomalies	4 (0.1%)	1.65 (0.62-4.41)	5 (0.1%)	0.60 (0.25-1.45)	10 (0.08%)	0.78 (0.42-1.45)
Certain conditions originating in perinatal period	0 (0.0%)	NA	2 (0.04%)	32.6 (8.14-130.2)	0 (0.0%)	NA
Complications of pregnancy, childbirth, puerperium	1 (0.02%)	1.92 (0.27-13.66)	2 (0.04%)	1.16 (0.29-4.63)	2 (0.02%)	0.82 (0.21-3.28)
Symptoms, signs and ill-defined conditions	18 (0.4%)	1.43 (0.90-2.27)	33 (0.6%)	0.83 (0.59-1.17)	115 (0.9%)	2.28 (1.90-2.73)
Other cause of death	217 (4.4%)	1.49 (1.30-1.70)	450 (8.4%)	1.01 (0.92-1.11)	1,382 (11.4%)	3.13 (2.97-3.30)

NA, not applicable.

Overall, the SMRs for non-cancer deaths decreased during the first 5 years post-diagnosis. Mortality risks increased with prolonged survival time and were highest after 5 years post thyroid cancer diagnosis, with an associated SMR of 2.26 (95% CI: 2.21–2.32). This increasing trend was observed for most causes of death and was highest for Alzheimer’s disease (5+ years: SMR: 8.27; 95% CI: 7.37–9.28). However, the SMR for suicide (1 year: SMR: 1.51; 95% CI: 1.04–2.20) as well as for death from aortic aneurysm and dissection (1 year: SMR: 3.66; 95% CI: 2.41–5.56) in the first post-diagnostic year were the highest compared with other timeframes during the entire follow-up period (**Table 3**).

### Mortality Rates for Patients With Thyroid Tumors

**Figure 2:**
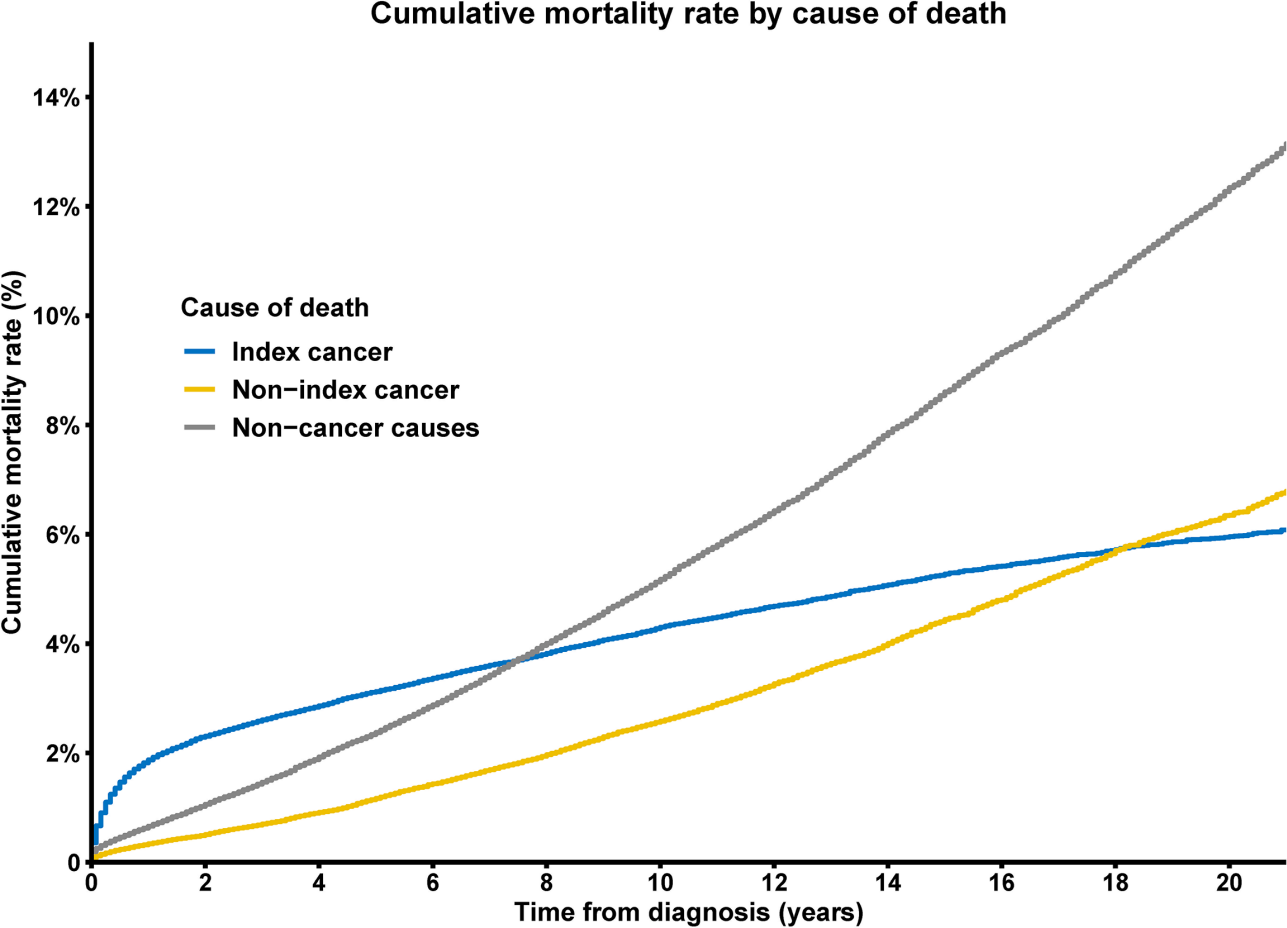
Cumulative mortality rate of deaths from index cancer, non-index cancer and other non-cancer causes among patients with thyroid cancer.

With all causes combined, the 1-year CMR for thyroid cancer patients was 2.2%, whereas the 5-year all-cause CMR was 5.9%, and the 10-year all-cause CMR was 11.0%. When performing cause-specific analyses, we observed that the CMR for thyroid cancer was higher than other for causes in the first few years after diagnosis, while the CMR for non-cancer causes of death overtook that of thyroid cancer, and non-cancer deaths were the most common approximately 8 years following cancer diagnosis (**Figure 2**).

**Figure 3:**
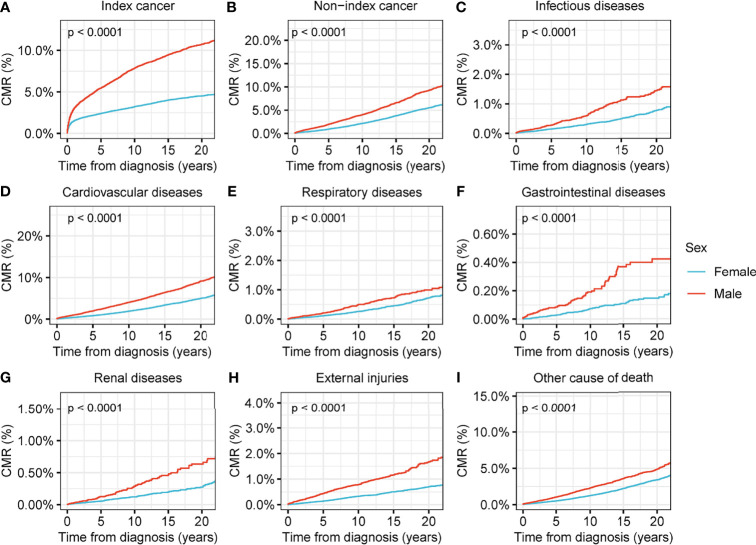
Cumulative mortality rate (CMR) among patients with thyroid cancer by sex. **(A)** CMR from index cancer among patients with thyroid cancer by sex. **(B)** CMR from non-index cancer among patients with thyroid cancer by sex. **(C)** CMR from infectious diseases among patients with thyroid cancer by sex. **(D)** CMR from cardiovascular diseases among patients with thyroid cancer by sex. **(E)** CMR from respiratory diseases among patients with thyroid cancer by sex. **(F)** CMR from gastrointestinal diseases among patients with thyroid cancer by sex. **(G)** CMR from renal diseases among patients with thyroid cancer by sex. **(H)** CMR from external injuries among patients with thyroid cancer by sex. **(I)** CMR from other non-cancer causes among patients with thyroid cancer by sex.

In addition, we conducted secondary CMR analyses using different variables to determine the subpopulations at higher risk of death among the evaluated thyroid cancer patients. We observed that, although the number of male patients diagnosed with thyroid malignancy was much lower than that of female patients (**Table 1**), males were under greater threat of death from nearly all causes compared with females (p<0.001 for all causes of death) (**Figure 3**). Older age was associated with a higher CMR for all causes of death (**Figure S3**), and black patients had higher a CMR for deaths from thyroid cancer (p<0.001), non-index cancers (p<0.001), infectious diseases (p=0.002), cardiovascular diseases (p<0.001), and renal diseases (p<0.001) compared with white patients. The variances between black and white patients in terms of the CMR for respiratory diseases (p=0.2), gastrointestinal diseases (p=0.8), or external injuries (p=0.5) showed no statistical significance (**Figure S4**). Advanced disease stages were associated with a significantly higher CMR for nearly all causes of death (**Figure S5**).

**Figure 4:**
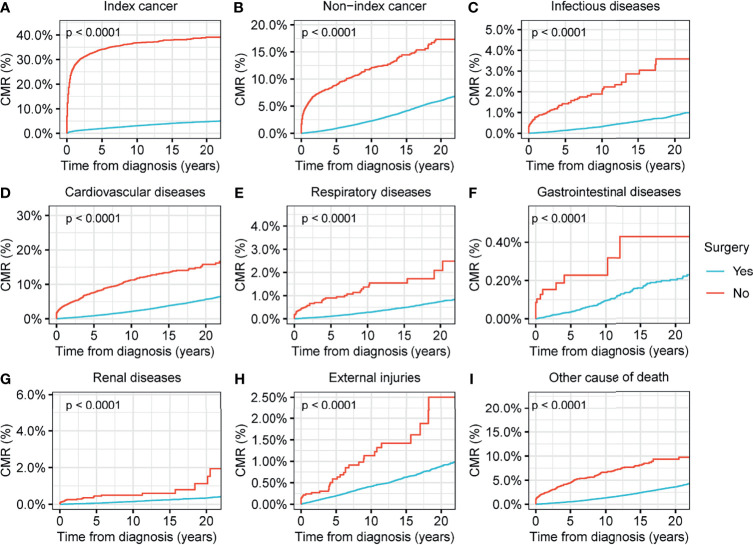
Cumulative mortality rate (CMR) among patients with thyroid cancer by surgery. **(A)** CMR from index cancer among patients with thyroid cancer by surgery. **(B)** CMR from non-index cancer among patients with thyroid cancer by surgery. **(C)** CMR from infectious diseases among patients with thyroid cancer by surgery. **(D)** CMR from cardiovascular diseases among patients with thyroid cancer by surgery. **(E)** CMR from respiratory diseases among patients with thyroid cancer by surgery. **(F)** CMR from gastrointestinal diseases among patients with thyroid cancer by surgery. **(G)** CMR from renal diseases among patients with thyroid cancer by surgery. **(H)** CMR from external injuries among patients with thyroid cancer by surgery. **(I)** CMR from other non-cancer causes among patients with thyroid cancer by surgery.

**Figure 5:**
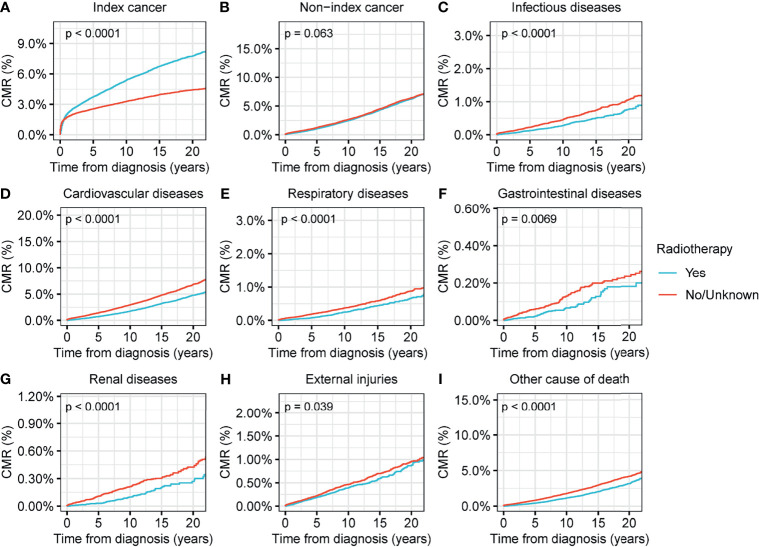
Cumulative mortality rate (CMR) among patients with thyroid cancer by radiotherapy. **(A)** CMR from index cancer among patients with thyroid cancer by radiotherapy. **(B)** CMR from non-index cancer among patients with thyroid cancer by radiotherapy. **(C)** CMR from infectious diseases among patients with thyroid cancer by radiotherapy. **(D)** CMR from cardiovascular diseases among patients with thyroid cancer by radiotherapy. **(E)** CMR from respiratory diseases among patients with thyroid cancer by radiotherapy. **(F)** CMR from gastrointestinal diseases among patients with thyroid cancer by radiotherapy. **(G)** CMR from renal diseases among patients with thyroid cancer by surgery. **(H)** CMR from external injuries among patients with thyroid cancer by radiotherapy. **(I)** CMR from other non-cancer causes among patients with thyroid cancer by radiotherapy.

We found that surgical interventions reduced the CMR for all causes of death in patients with thyroid malignancy (p<0.001) (**Figure 4**). Radiotherapy reduced the CMR for nearly all causes of deaths in thyroid malignancy, with the exception of non-index cancers (**Figure 5**). Comparisons between patients who underwent radiotherapy and those who did not showed no statistically significant differences in variances in the CMR (p=0.06) (**Figure 5**). CMR analyses with respect to chemotherapy were not performed in the current study because of the low prevalence of this intervention (**Table 1**).

## Discussion

In the current research, we summarized the spectrum of causes of death amongst approximately 183,000 US patients with thyroid carcinoma. The research has revealed that the proportion of non-cancer deaths occurring in patients with thyroid carcinoma exceeded the proportion of index-cancer deaths (i.e., deaths from thyroid malignancy), and that the risk of mortality from non-cancer comorbidities was nearly 1.6-fold that of the cancer-free population.

We have demonstrated that non-cancer comorbidities may be the dominant cause of death in thyroid cancer patients, as has been previously reported for other cancers. More and more research has emphasized the importance of non-cancer comorbidities in the healthcare of patients with cancer (7, 8, 31, 32). In certain types of malignancy, including breast malignancy (31), colorectal malignancy (8), and prostate malignancy (32), non-cancer comorbidities have become the dominant cause of death in recent decades. This research adds to this finding. This important role of non-cancer deaths in thyroid cancer patients could partly be interpreted by the relatively favorable prognosis for differentiated thyroid carcinoma (33). The reduction in the index-cancer deaths in thyroid cancer should be attributed to the tremendous advances achieved in the detection and treatments of this disease, even over-diagnosis and over-diagnosis in some low-risk cases (34). Recently, personalized medicine, in which a person’s prevention, screening, and treatment are optimized by more effective strategies based on genetic information and other personalized characteristics, become a new trend in the management of thyroid carcinomas (33, 35). The management of thyroid carcinomas switch from “one size fits all” therapeutic measures that included total thyroidectomy, radioiodine and TSH suppression, to much more personalized strategies that currently include lobectomy or just active surveillance for low-risk of recurrence thyroid patients (33, 35). In addition, personalized medicine provides new directions for treatment of non-cancer comorbidities in thyroid cancer patients, because the non-cancer comorbidities are also highly individualized and might be distinct across patients.

Our results demonstrate that the risk of nearly all types of non-cancer comorbidities increases with prolonged follow-up time. Advances in early detection have led to an increasing proportion of patients diagnosed at an early stage. Our data showed that only 4% of thyroid malignancies were diagnosed with a distant metastatic tumor. The high early detection rate and generally favorable prognosis for this disease contribute to the increasing prevalence of long-term thyroid cancer survivors. This emphasized the importance of uncovering the detailed causes of death in these long-term malignancy patients, as a cancer history will meaningfully alter the distribution of causes of death among these individuals and will inform interventions with respect to mitigating mortality risk and promoting survivorship and quality of life (7, 8, 31, 32). Our work, as well as that of prior studies, shows that cancer and its treatments may increase the short- and long-term risks of non-cancer death (7, 15).

Cardiovascular disease was responsible for more than one-fifth of all deaths among thyroid cancer patients in this study, making it a significant cause of death among patients with thyroid carcinoma. Earlier findings and cutting-edge treatment guidelines indicated that receiving radioactive iodine for thyroid cancer is related to an elevated incidence of cardiovascular disease, and persistent thyroxine exposure and/or thyroid-stimulating hormone restraining therapy increase the risk of cardiovascular-specific mortality, by impairing large arteries, stiffening small arteries (accepted as a surrogate marker for cardiovascular disease), and leading to hypertension and/or cardiac arrhythmias (36–41). In addition, thyroid cancer patients often receive doses of thyroid hormones that are slightly higher than their daily needs (i.e., suppressive thyroid hormone therapy) in an endeavor to decrease the development of any thyroid malignancy cells following the preliminary course of treatment. We acknowledge that patients who receive high doses of thyroid hormone replacement for a long time are at increased risk of bone loss and heart rhythm changes (i.e., atrial fibrillation) (38).

In this study, we observed a remarkably elevated mortality risk due to hypertension in patients with thyroid carcinoma. Previous research has likewise uncovered a high incidence of hypertension among patients with thyroid carcinoma (23). Patients with thyroid dysfunction (both hypothyroidism and hyperthyroidism) also show an increased prevalence of hypertension (42, 43), and some novel thyroid cancer therapies may likewise add to the possibility of developing hypertension in patients with thyroid malignancy. For example, levantinib is a newly developed molecularly targeted agent for iodine-refractory differentiated thyroid cancer (44), and studies to date demonstrate that most of the patients treated with levantinib had concomitant hypertension (45).

Although thyroid cancer was diagnosed less frequently in male patients, we observed a higher rate of mortality from nearly all causes in male patients with thyroid malignancy in this study. Studies have demonstrated that thyroid malignancy characteristically presents at a more advanced stage and there is a worse tumor prognosis in males (46, 47). Research on the influence of sex hormones on thyroid malignancy has continued to be inconclusive, while plenty of experimental research studies have indicated that estrogen and other hormones and their receptors may lead to tumorigenesis and tumor progression (46, 47).

Interestingly, we discovered that the incidence of suicide was highest within the first post-diagnostic year, consistent with previous pan-cancer studies (14, 26). Prior studies have suggested that suicide risk varies according to the malignant potential of the cancer (26). Our results indicate that thyroid cancer, whose prognosis is more favorable, are also associated with an elevated risk of suicide. External injuries (i.e., mainly due to accidents and suicides) were the third leading non-cancer cause of death in our study, suggesting the great importance of ensuring the safety of thyroid cancer survivors and to avoid the occurrence of external injuries in this population.

We found a remarkably increased risk of mortality from Alzheimer’s diseases in long-term thyroid malignancy patients. The thyroid gland plays crucial role in the regulation of metabolism, growth, and development of various tissues, organs, systems, including the central nervous system (48). Accumulating evidence has demonstrated the importance of thyroid function in the development of Alzheimer’s disease, while Alzheimer’s disease also leads to a significant increase in the morbidity of thyroid dysfunction (49). Although many studies have confirmed that thyroid function is significantly associated with cognitive impairment, the underlying mechanisms are not well understood (49). Some studies hypothesized that the crucial role that thyroid function plays in the pathogenesis of Alzheimer’s disease is achieved by affecting the metabolism of Aβ and p-tau (48, 49). Hyperthyroidism as well as hypothyroidism can induce hippocampus degeneration and impair cognition through a reduction in long-term potentiation (48–52). The relationships between thyroid function and Alzheimer’s disease provide new therapeutic strategies for Alzheimer’s disease. Understanding the underlying mechanisms will help in providing novel approaches for the management of patients with Alzheimer’s disease. Our results demonstrate that thyroid malignancy, as a major type of thyroid dysfunction, might also be related to the long-term possibility of Alzheimer’s disease. We conclude that this information should be common knowledge (gained through continuing education) among clinicians and researchers, and targeted interventions should be applied aggressively and consistently in order to mitigate this risk.

Our study had several limitations. First, the SEER 2018 database lacks information on patient comorbidities and family history, as well as detailed information on therapy, all of which may have an impact on thyroid cancer prognoses. Therefore, deeper and more comprehensive analyses, including risk stratification and the assessment of the impact of the aforementioned factors were impossible to conduct herein. Second, there is a possibility of misclassification with respect to cause of death that is inherent to epidemiologic research, and there is some reporting bias associated with death certificates. However, the SEER database uses a systematic and standard data collection procedure in order to ensure the accuracy of cause of death determinations (12), thereby substantially mitigating the possible impacts of these potential biases on study results and interpretation. Nevertheless, the strengths of the current study were the large-scale study cohort and the detailed cause-of-death records, which enabled us to comprehensively analyze the cause of death among patients with thyroid malignancy. Our study can provide guidance for future research, as well as guide clinicians to achieve better long-term outcomes in patients with thyroid carcinoma.

## Conclusion

Non-cancer causes of death have replaced primary index malignancy as the main cause of death amongst patients with thyroid carcinoma in the US. We clarified that patients with thyroid carcinoma have a higher risk of non-cancer deaths than the cancer-free population. As the survival time of patients with thyroid carcinoma increases, non-cancer deaths will increase in this population and the relative risk of mortality from various causes will increase exponentially. Hence, clinicians should practice careful chronic disease management for patients with thyroid malignancy. In the early phase following malignancy diagnosis, it is necessary to provide appropriate and comprehensive education and psychological counseling to patients in order to prevent the occurrence of depression and suicide. As the survival period is extended, attention should be paid to lifestyle improvements and the rational use of tumor drugs to prevent Alzheimer’s disease and other non-cancer diseases. Our findings can guide the direction of future research studies and inform future medical guidelines.

## Data Availability Statement

Publicly available datasets were analyzed in this study. This data can be found here: https://seer.cancer.gov/.

## Author Contributions

Research designer: HL and YZ. Collecting, analyzing and interpreting data: YZ, QW, ZZ, HL and JN. The main contributors to writing manuscripts: HL, QW, ZZ, HL and JN. The final draft read and approved by all authors.

## Conflict of Interest

The authors declare that the research was conducted in the absence of any commercial or financial relationships that could be construed as a potential conflict of interest.

## Publisher’s Note

All claims expressed in this article are solely those of the authors and do not necessarily represent those of their affiliated organizations, or those of the publisher, the editors and the reviewers. Any product that may be evaluated in this article, or claim that may be made by its manufacturer, is not guaranteed or endorsed by the publisher.
