# Supplementary material for: Cause of Death Among Patients With Thyroid Cancer: A Population-Based Study
Source: Front Oncol. 2022 Mar 14;12:852347. doi: 10.3389/fonc.2022.852347 (PMC8964038; doi:10.3389/fonc.2022.852347)
Supplement: Supplementary file 1 [file DataSheet_1.docx]

**Supplementary Figures**

**Figure S1** Inclusion and exclusion criteria for the patients in this study.

**Figure S2** Trends in cause of death among patients with thyroid cancer from 1975 to 2016.

**Figure S3** Cumulative mortality rate (CMR) among patients with thyroid cancer by age at cancer diagnosis. (A) CMR from index cancer among patients with thyroid cancer by age at cancer diagnosis. (B) CMR from non-index cancer among patients with thyroid cancer by age at cancer diagnosis. (C) CMR from infectious diseases among patients with thyroid cancer by age at cancer diagnosis. (D) CMR from cardiovascular diseases among patients with thyroid cancer by age at cancer diagnosis. (E) CMR from respiratory diseases among patients with thyroid cancer by age at cancer diagnosis. (F) CMR from gastrointestinal diseases among patients with thyroid cancer by age at cancer diagnosis. (G) CMR from renal diseases among patients with thyroid cancer by age at cancer diagnosis. (H) CMR from external injuries among patients with thyroid cancer by age at cancer diagnosis. (I) CMR from other non-cancer causes among patients with thyroid cancer by age at cancer diagnosis.

**Figure S4** Cumulative mortality rate (CMR) among patients with thyroid cancer by race. (A) CMR from index cancer among patients with thyroid cancer by race. (B) CMR from non-index cancer among patients with thyroid cancer by race. (C) CMR from infectious diseases among patients with thyroid cancer by race. (D) CMR from cardiovascular diseases among patients with thyroid cancer by race. (E) CMR from respiratory diseases among patients with thyroid cancer by race. (F) CMR from gastrointestinal diseases among patients with thyroid cancer by race. (G) CMR from renal diseases among patients with thyroid cancer by race. (H) CMR from external injuries among patients with thyroid cancer by race. (I) CMR from other non-cancer causes among patients with thyroid cancer by race.

**Figure S5** Cumulative mortality rate (CMR) among patients with thyroid cancer by stage. (A) CMR from index cancer among patients with thyroid cancer by stage. (B) CMR from non-index cancer among patients with thyroid cancer by stage. (C) CMR from infectious diseases among patients with thyroid cancer by stage. (D) CMR from cardiovascular diseases among patients with thyroid cancer by stage. (E) CMR from respiratory diseases among patients with thyroid cancer by stage. (F) CMR from gastrointestinal diseases among patients with thyroid cancer by stage. (G) CMR from renal diseases among patients with thyroid cancer by stage. (H) CMR from external injuries among patients with thyroid cancer by stage. (I) CMR from other non-cancer causes among patients with thyroid cancer by stage.


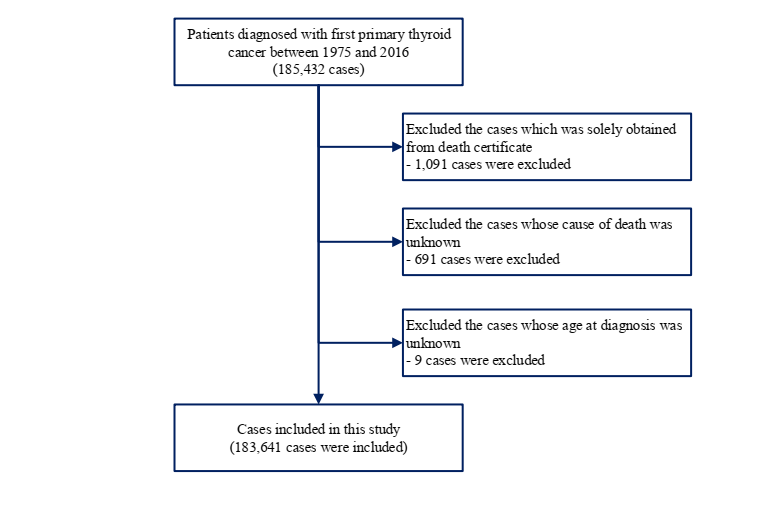


**Figure S1** Inclusion and exclusion criteria for the patients in this study.


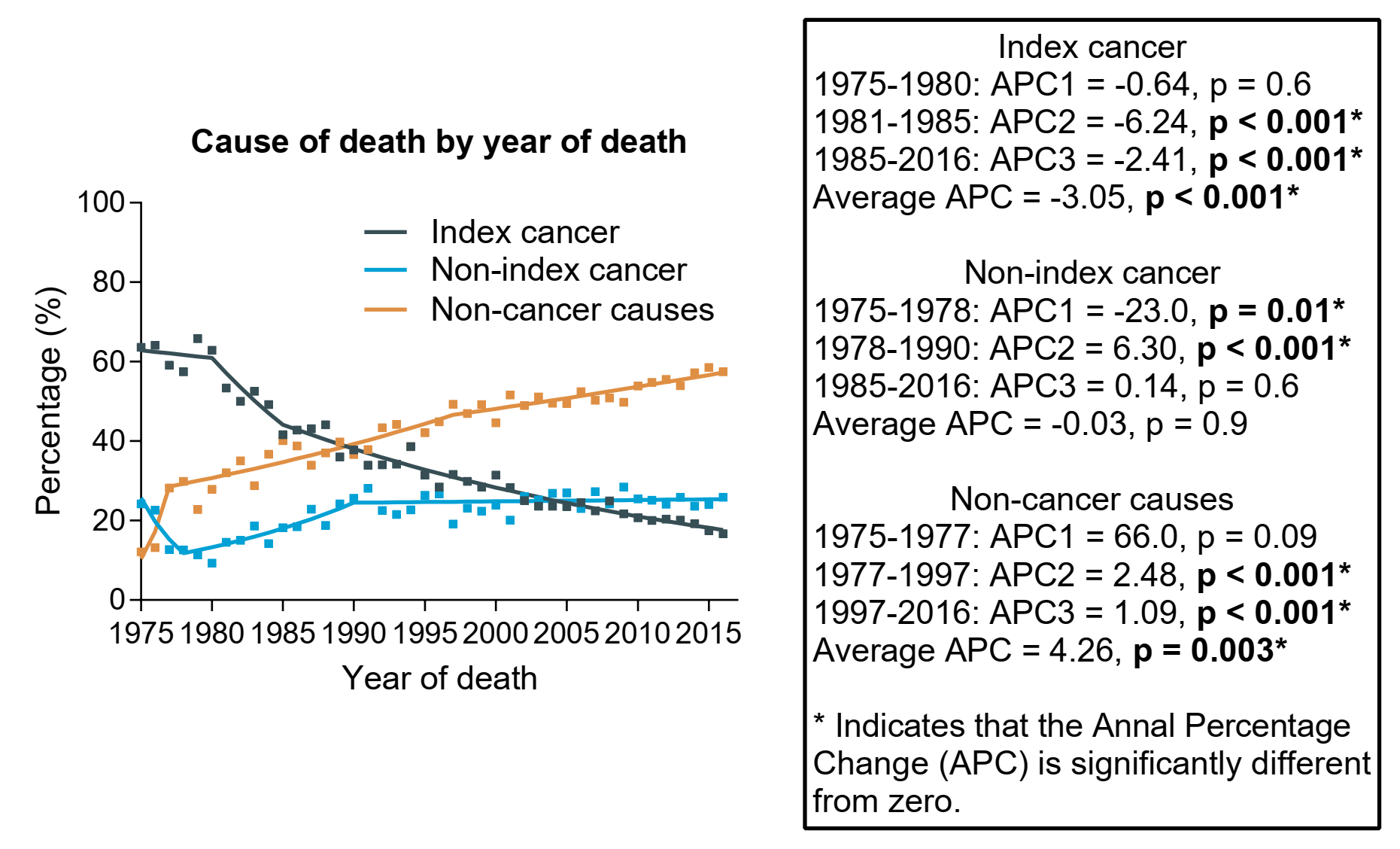
 **Figure S2** Trends in cause of death among patients with thyroid cancer from 1975 to 2016.


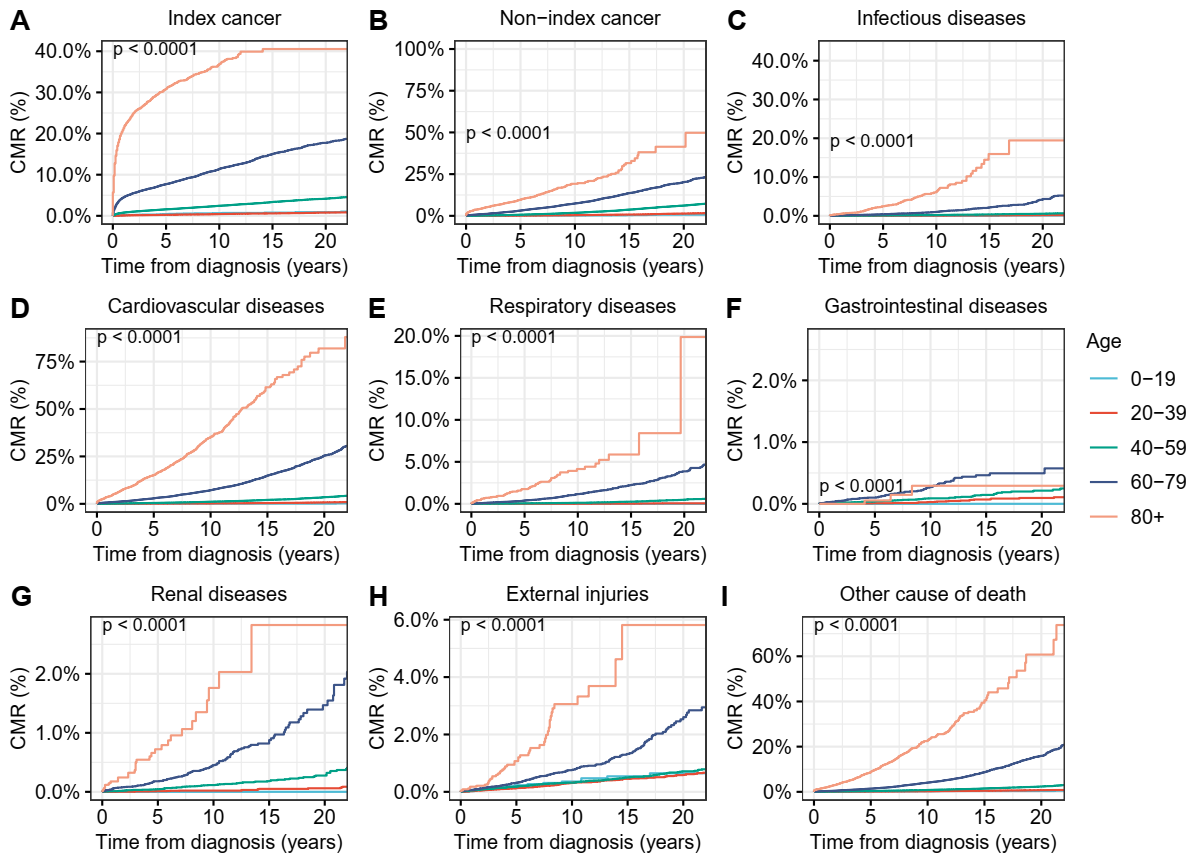


**Figure S3** Cumulative mortality rate (CMR) among patients with thyroid cancer by age at cancer diagnosis. (A) CMR from index cancer among patients with thyroid cancer by age at cancer diagnosis. (B) CMR from non-index cancer among patients with thyroid cancer by age at cancer diagnosis. (C) CMR from infectious diseases among patients with thyroid cancer by age at cancer diagnosis. (D) CMR from cardiovascular diseases among patients with thyroid cancer by age at cancer diagnosis. (E) CMR from respiratory diseases among patients with thyroid cancer by age at cancer diagnosis. (F) CMR from gastrointestinal diseases among patients with thyroid cancer by age at cancer diagnosis. (G) CMR from renal diseases among patients with thyroid cancer by age at cancer diagnosis. (H) CMR from external injuries among patients with thyroid cancer by age at cancer diagnosis. (I) CMR from other non-cancer causes among patients with thyroid cancer by age at cancer diagnosis.


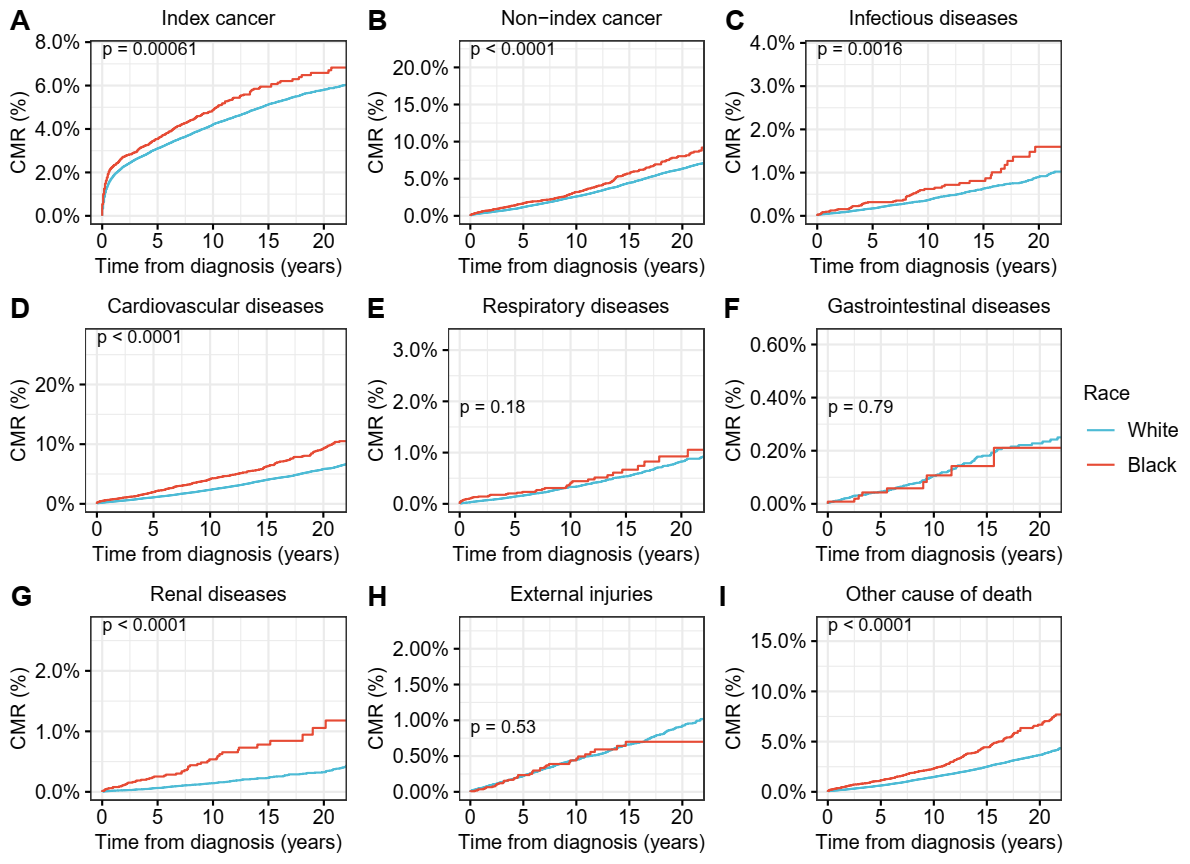


**Figure S4** Cumulative mortality rate (CMR) among patients with thyroid cancer by race. (A) CMR from index cancer among patients with thyroid cancer by race. (B) CMR from non-index cancer among patients with thyroid cancer by race. (C) CMR from infectious diseases among patients with thyroid cancer by race. (D) CMR from cardiovascular diseases among patients with thyroid cancer by race. (E) CMR from respiratory diseases among patients with thyroid cancer by race. (F) CMR from gastrointestinal diseases among patients with thyroid cancer by race. (G) CMR from renal diseases among patients with thyroid cancer by race. (H) CMR from external injuries among patients with thyroid cancer by race. (I) CMR from other non-cancer causes among patients with thyroid cancer by race.


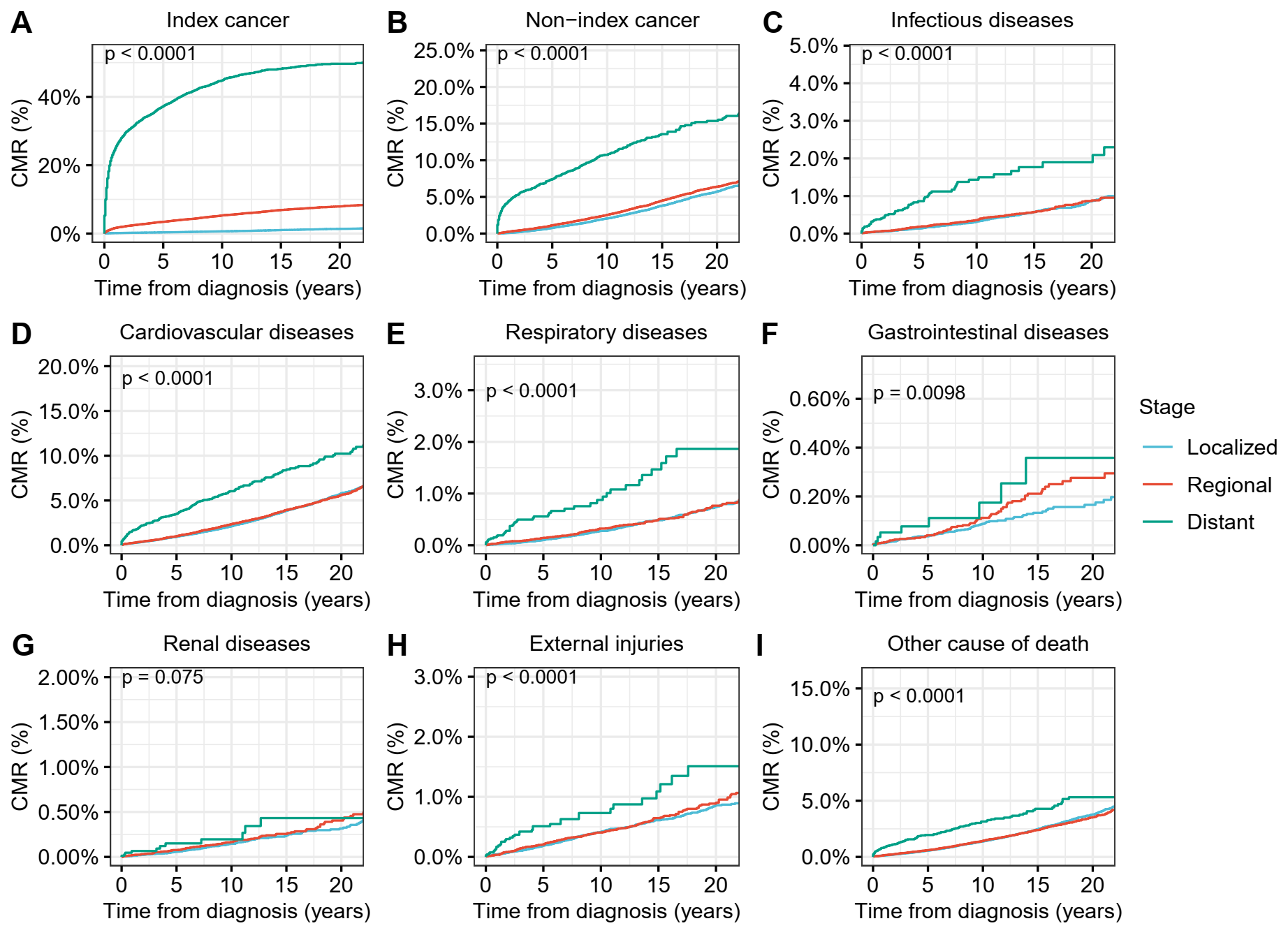


**Figure S5** Cumulative mortality rate (CMR) among patients with thyroid cancer by stage. (A) CMR from index cancer among patients with thyroid cancer by stage. (B) CMR from non-index cancer among patients with thyroid cancer by stage. (C) CMR from infectious diseases among patients with thyroid cancer by stage. (D) CMR from cardiovascular diseases among patients with thyroid cancer by stage. (E) CMR from respiratory diseases among patients with thyroid cancer by stage. (F) CMR from gastrointestinal diseases among patients with thyroid cancer by stage. (G) CMR from renal diseases among patients with thyroid cancer by stage. (H) CMR from external injuries among patients with thyroid cancer by stage. (I) CMR from other non-cancer causes among patients with thyroid cancer by stage.
